# Highlight: Footprints of Male Pregnancy and Sexual Selection in the Genomes of Seahorses and Pipefish

**DOI:** 10.1093/gbe/evaf122

**Published:** 2025-07-10

**Authors:** Pedro Andrade

We may take it for granted, but in many ways, it remains puzzling why males and females of many species are so different, despite sharing the majority of their genome. Understanding the evolution of sexual traits and sex determination depends in large part on our understanding of how organisms resolve sexual antagonism—the conflict that arises when interactions between alleles (at one locus, or between different loci) have opposing fitness effects in males and females for a shared trait (Bonduriansky and Chenoweth 2009). Intralocus sexual antagonism, a form of balancing selection, can be mitigated by sex-specific expression and ultimately resolved by the co-localization of such loci to chromosomes with sex-biased expression. This makes sexual antagonism and sex determination interlinked in shaping the evolution of genomes.

**Fig. 1.**
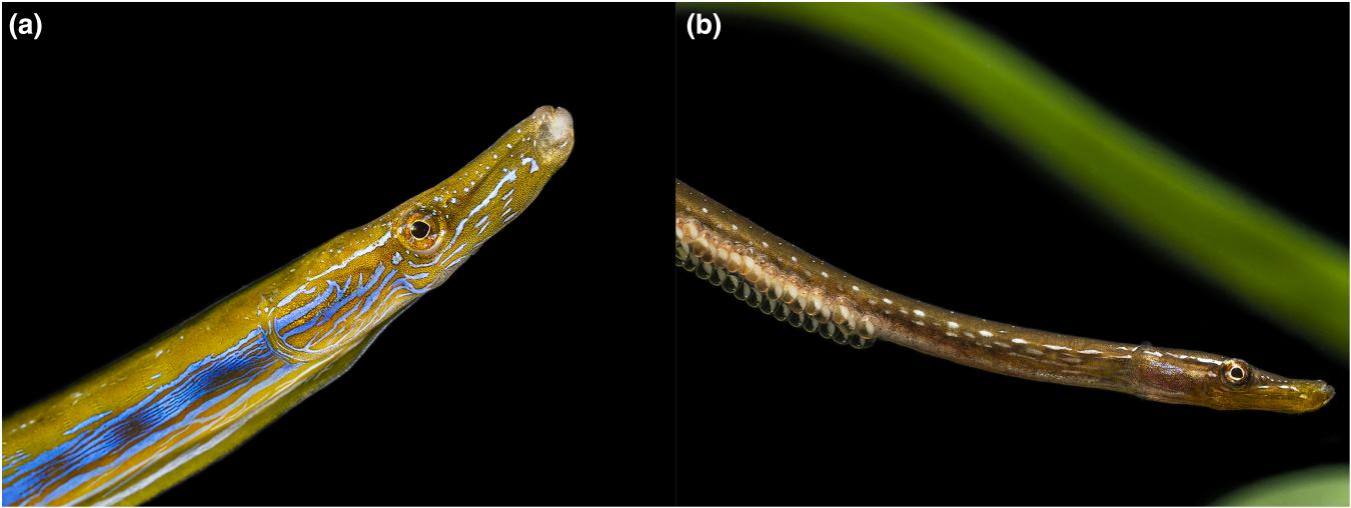
Precopulatory sexual selection and male pregnancy in the straightnose pipefish (*Nerophis ophidion*), one of the species studied by Dubin et al. (2025). In the straightnose pipefish, females a) are larger and more ornamented than males b), who carry their eggs externally on their ventral side. Image credit: Olivia Roth.

Olivia Roth—principal investigator at the Christian-Albrechts University in Kiel, Germany—advocates for an approach to decipher the links between sexual antagonism and sex determination: “If one aims to gain a deep understanding of particular evolutionary phenomena, one should move towards the unconventional,” Roth says. To her, this meant working with syngnathids (i.e. seahorses and pipefish; Fig. 1) as unconventional models due to their reproductive strategies, as these fish feature “a unique evolution of male pregnancy coupled with a convergent evolution of sex role reversal and brooding structures.” In species of teleost fish that have parental care, paternal care is more common; yet sex-role reversal (i.e. when females compete more intensely than males for access to mates) is not frequently observed, because the male reproductive rate still typically exceeds the female rate (Eens and Pinxten 2000). Traditionally, it was thought that high-investment paternal care (i.e. male pregnancy) is what drove sex-role reversal in syngnathids, but current evidence suggests these traits are decoupled. This is because, while male pregnancy is widespread in this group, it coincides with a large variation in the degree of sex-role reversal across species.

In their new article in *Genome Biology and Evolution* (Dubin et al. 2025), Roth's team focused on three syngnathid species—the seahorse *Hippocampus erectus* and the pipefish species *Nerophis ophidion* and *Syngnathus typhle*—in which the degree of development of male pregnancy and the intensity of precopulatory sexual selection were decoupled. The authors then queried their transcriptomes and genomes for differences between the sexes. Their objective was to test if differences in male pregnancy and precopulatory sexual selection, which drive phenotypic differences between the sexes, were associated with signatures of sexual antagonism and therefore potentially linked to the evolution of sex determination mechanisms.

After assembling two novel pipefish genomes to aid their study, the authors used RNA-sequencing data of males and females of each of the three species to identify differentially expressed alleles between sexes. Among the three species, the one with the highest sex bias in expression was the seahorse, in which highly male-biased genes predominated and co-localized for the most part to the same genomic region. This region was highly differentiated in female–male genome scans, which is indicative of a putative XX/XY sex determination system and well-resolved sexual antagonism in this species. The authors also found sex-biased expression for the two pipefish species, but the differences were similarly distributed between males and females. In these two species, loci involved in female–male differentiation are scattered throughout the genome, suggesting that no single sex-determining chromosome or region exists in these species. These general patterns also emerged when the researchers investigated whether there was an overlap between highly differentiated genes and intersex expression bias—there is, and their number reflected the strength of the signal for a single sex determination system. The seahorse had the highest overlap, while the pipefish *S. typhle* had the lowest, and *N. ophidion* had an intermediate pattern of differentiation scattered throughout the genome.

While these results could be interpreted as a simple gradient of sexual antagonism driven by differences in the strength of precopulatory sexual selection and male pregnancy, the reproductive biology of these species suggests more complex interactions. In syngnathids, sex role reversal is not predicted by male pregnancy, but rather by mating patterns, since monogamous species have conventional sex roles, while those with reverse sex roles tend to be polygamous (Wilson et al. 2003). The seahorse's conventional sexual selection regime (*H. erectus* is a monogamous species in which larger males may compete for females) seems to favor the resolution of sexual antagonism by tying it to the evolution of an XY sex determination system, even in the face of well-developed male pregnancy. *Nerophis ophidion*, the pipefish species with moderate genomic signatures of sexual antagonism, has the most basic form of male pregnancy and extreme reversed precopulatory sexual selection (Fig. 1). Thus, male pregnancy alone does not account for the signatures of sexual antagonism in the genomes of syngnathids. Dubin et al. argue that these effects are likely driven by interactions between precopulatory sexual selection and male pregnancy, since male choice of ornamented females in the two pipefish species seems to be a determining differentiator of the strength of sexual selection between them.

The authors got an additional surprise when combing through their data. A previous study (Long et al. 2023) had already identified a putative homomorphic sex chromosome pair in *H. erectus* seahorses; however, the candidate sex chromosome pair identified by Dubin et al. was not the one found by the previous group. These differences likely reflect each group's use of different seahorse breeds, hinting at surprising variation in sex determination mechanisms within a species. Albeit surprising, these results are in line with previously described high sex chromosome turnover in teleost fish, as well as the complex evolutionary dynamics of sex determination of syngnathids suggested by Dubin et al. (2025). Roth adds: “That XX/XY has evolved in *Hippocampus*, while in other syngnathids sex determination has taken different evolutionary pathways, is a very important finding, as it will allow us to test hypotheses generated about sexual dimorphism in the future.”

Roth believes much of the strength of this research goes beyond evolutionary genomics: “Science is heavily biased towards mammals, and thus having XX/XY is, by the public, often perceived as a given despite that sex determination is very diverse across animals”. Like other researchers, Roth is aware of how findings in our field have implications for how the public perceives nature and, ultimately, ourselves, as she concludes: “This can also help in providing a better understanding and acceptance of non-binary gender identities in our community and may support the need for developing non-gender-biased diagnosis and treatment for improving public health.”

*Want to learn more?* Check out these other articles on sexual antagonism and sex determination recently published in *Genome Biology and Evolution*:

Barata C, Snook RR, Ritchie MG, Kosiol C. Selection on the fly: short-term adaptation to an altered sexual selection regime in *Drosophila pseudoobscura*. Genome Biol Evol. 2023:15(7):evad113. https://doi.org/10.1093/gbe/evad113.
Mishra P, Barrera TS, Grieshop K, Agrawal AF. *Cis*-regulatory variation in relation to sex and sexual dimorphism in *Drosophila melanogaster*. Genome Biol Evol. 2024:16(11):evae234. https://doi.org/10.1093/gbe/evae234.
Palmer Droguett DH, Fletcher M, Alston BT, Kocher S, Cabral-de-Mello DC, Wright AE. Neo-sex chromosome evolution in treehoppers despite long-term X chromosome conservation. Genome Biol Evol. 2024:16(12):evae264. https://doi.org/10.1093/gbe/evae264.
